# Effective Treatment of Empyema With Fistula Using Saline Irrigation Through Tube Thoracostomy: A Case Report

**DOI:** 10.7759/cureus.86386

**Published:** 2025-06-19

**Authors:** Ryokan Ikebe, Mizuho Namiki, Munekazu Takeda, Takuya Oshiro, Shusuke Mori

**Affiliations:** 1 Department of Critical Care and Emergency Medicine, Tokyo Women's Medical University, Tokyo, JPN

**Keywords:** chest tube drainage, empyema with fistula, hemothorax complication, intrapleural lavage, non-surgical management, pleural infection, pulmonary embolism

## Abstract

Empyema with fistula formation represents a particularly challenging clinical entity, as the presence of an abnormal connection between the pleural cavity and the bronchial tree or lung parenchyma often exacerbates infection, hinders effective drainage, and complicates treatment. We report the case of a 66-year-old man who developed a massive pulmonary embolism, complicated by hemothorax and subsequent empyema with fistula formation, manifested by pneumothorax and purulent drainage. Despite the administration of anticoagulation and broad-spectrum antibiotics, the infection persisted, and surgical intervention was deemed too risky due to ongoing cardiopulmonary instability and residual pulmonary vascular obstruction. Given the high surgical risk, we initiated intrathoracic saline irrigation via a double-lumen irrigation suction tube as a conservative alternative. This strategy led to a prompt resolution of high fever and notable improvements in inflammatory markers. Saline lavage was administered over approximately one month, with progressive volume escalation and close clinical monitoring, ultimately resulting in radiological and clinical resolution of the empyema and closure of the fistula without serious complications. This case highlights the potential utility of intrapleural saline irrigation as a safe and effective therapeutic option in patients with fistulous empyema who are not suitable candidates for surgery. Careful patient selection and multidisciplinary collaboration are essential to ensure favorable outcomes in such complex scenarios.

## Introduction

Empyema, the accumulation of pus in the pleural space, remains a serious complication of pneumonia, trauma, or thoracic surgery [1]. The presence of a fistula, an abnormal communication between the pleural space and the bronchial tree or lung parenchyma, further complicates management by perpetuating infection and impeding healing [2]. Standard treatment typically involves prolonged antimicrobial therapy and effective pleural drainage, but these measures alone are often insufficient in the presence of a fistula [3]. Surgical interventions such as video-assisted thoracoscopic surgery (VATS), open window thoracostomy (OWT), or decortication are frequently required to achieve source control and promote healing [4].

In recent years, adjunctive intrapleural therapies, including fibrinolytics and saline irrigation, have emerged, aimed at enhancing pleural fluid evacuation and reducing the need for surgery [3]. The Pleural Irrigation Trial (PIT) demonstrated that saline irrigation could improve outcomes in pleural infections by facilitating drainage and reducing surgical referrals [5]. However, the application of intrapleural irrigation in the setting of a fistula is controversial due to the risk of introducing infection into the lung parenchyma and precipitating pneumonia [6]. The optimal irrigation protocol, including the volume and frequency of saline administration, remains undefined, and evidence is largely limited to case reports and small series [7].

In the context of empyema with fistula, the risk-benefit balance of intrapleural interventions must be carefully weighed, particularly in patients with significant comorbidities or contraindications to surgery. Pulmonary embolism, for instance, complicates both the clinical course and therapeutic options, as anticoagulation increases bleeding risk and may preclude invasive procedures [8]. Furthermore, patients with recent cardiac arrest or those who have undergone extracorporeal membrane oxygenation (ECMO) may be poor surgical candidates due to compromised cardiopulmonary reserve [9].

We present a case of fistulous empyema in a patient with massive pulmonary embolism and recent cardiac arrest, in whom surgical intervention was not feasible. The successful use of intrapleural saline irrigation in this context offers insight into a potential alternative strategy for managing complex empyema when standard approaches are contraindicated. This report aims to contribute to the evolving discussion on the role of pleural irrigation in challenging clinical scenarios and to provide practical considerations for its implementation.

## Case presentation

**Figure 1:**
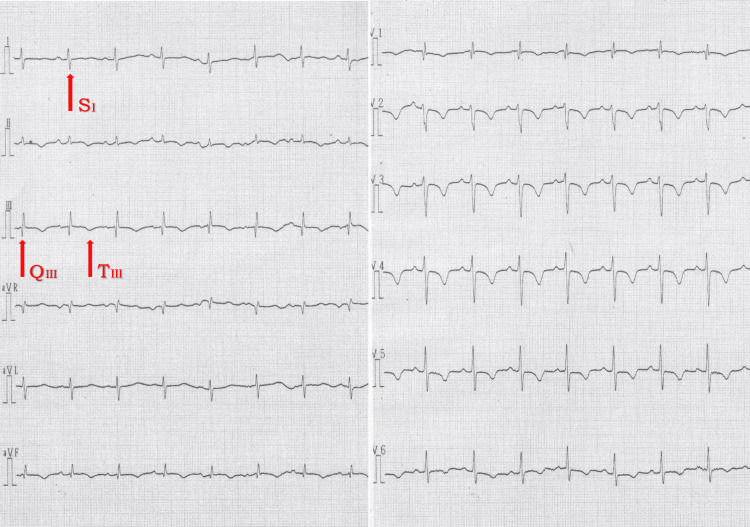
12-lead ECG on admission The ECG demonstrates the classic S1Q3T3 pattern, consisting of a prominent S wave in lead I, a Q wave in lead III, and a T wave inversion in lead III. This pattern is suggestive of acute right ventricular strain, often secondary to a massive pulmonary embolism. Although S1Q3T3 is not highly sensitive or specific, it remains a useful clinical clue when correlated with symptoms such as dyspnea and chest pain. Additional findings in this case included right axis deviation, further supporting the diagnosis of acute pulmonary embolism with right heart involvement. ECG, electrocardiogram

A 66-year-old man presented to the emergency department with dyspnea and chest pain. His medical history included bipolar disorder and prolonged immobility due to being bedridden after a bleeding duodenal ulcer. On arrival, his vital signs were as follows: blood pressure 125/82 mmHg, heart rate 98 beats per minute, respiratory rate 44 breaths per minute, and oxygen saturation 92% on 10 L/min of oxygen via a reservoir mask. The electrocardiogram (ECG) showed an S1Q3T3 pattern (Figure 1).

Transthoracic echocardiography at presentation demonstrated marked right ventricular enlargement, septal flattening forming a D-shaped left ventricle, and elevated tricuspid regurgitation velocity (3.3 m/s), all indicative of acute right heart strain due to pulmonary embolism. Laboratory analysis revealed an elevated D-dimer of 8.7 μg/mL (Table 1).

**Table 1:** Laboratory results on admission AG: anion gap, Lac: lactate, WBC: white blood cell, Neut: neutrophil, RBC: red blood cell, Hb: hemoglobin, Ht: hematocrit, Plt: platelet, PT: prothrombin time, APTT: activated partial thromboplastin time, AT-III: antithrombin III, FDP: fibrin degradation products, TAT: thrombin-antithrombin complex, TP: total protein, CK: creatine kinase, CK-MB: creatine kinase MB isoenzyme, BUN: blood urea nitrogen, Cre: creatinine, CRP: C-reactive protein

	Value	Reference range	Unit
Arterial Blood Gas			
pH	7.451	7.35-7.45	
pCO_2_	21.4	35-45	mmHg
pO_2_	63.3	80-100	mmHg
HCO_3_^-^	14.6	22-26	mmol/L
AG	18.3	8-12	mEq/L
Lac	4.5	0.5-2.2	mmol/L
Complete Blood Count			
WBC	8.77	4.00-8.60	×10³/μL
Neut	77.3	38-71	%
RBC	5.39	4.1-5.3	×10^6^/μL
Hb	14.4	14-18	g/dL
Ht	45.9	39-47	%
Plt	21.5	15-35	×10⁴/μL
Coagulation Profile			
PT	15.3	10-13	second
APTT	54.9	25.5-37.5	second
AT-III	96	80-130	%
FDP	36.9	<5.0	μg/mL
D-dimer	8.7	<1.0	μg/mL
TAT	22.1	<4.0	ng/mL
Fibrinogen	344	145-350	mg/dL
Biochemistry			
TP	7.3	6.5-8.2	g/dL
Albumin	3.5	3.8-5.1	g/dL
CK	114	62-287	U/L
CK-MB	6	<13	U/L
BUN	19.2	8.0-20.0	mg/dL
Cre	1.04	0.69-1.06	mg/dL
Na^+^	139	135-145	mEq/L
K^+^	5.6	3.4-4.9	mEq/L
Cl^-^	108	98-108	mEq/L
Ca^+^	8.8	8.5-9.9	mg/dL
Glucose	167	75-109	mg/dL
CRP	4.17	<0.33	mg/dL
Procalcitonin	0.04	<0.05	ng/mL

**Figure 2:**
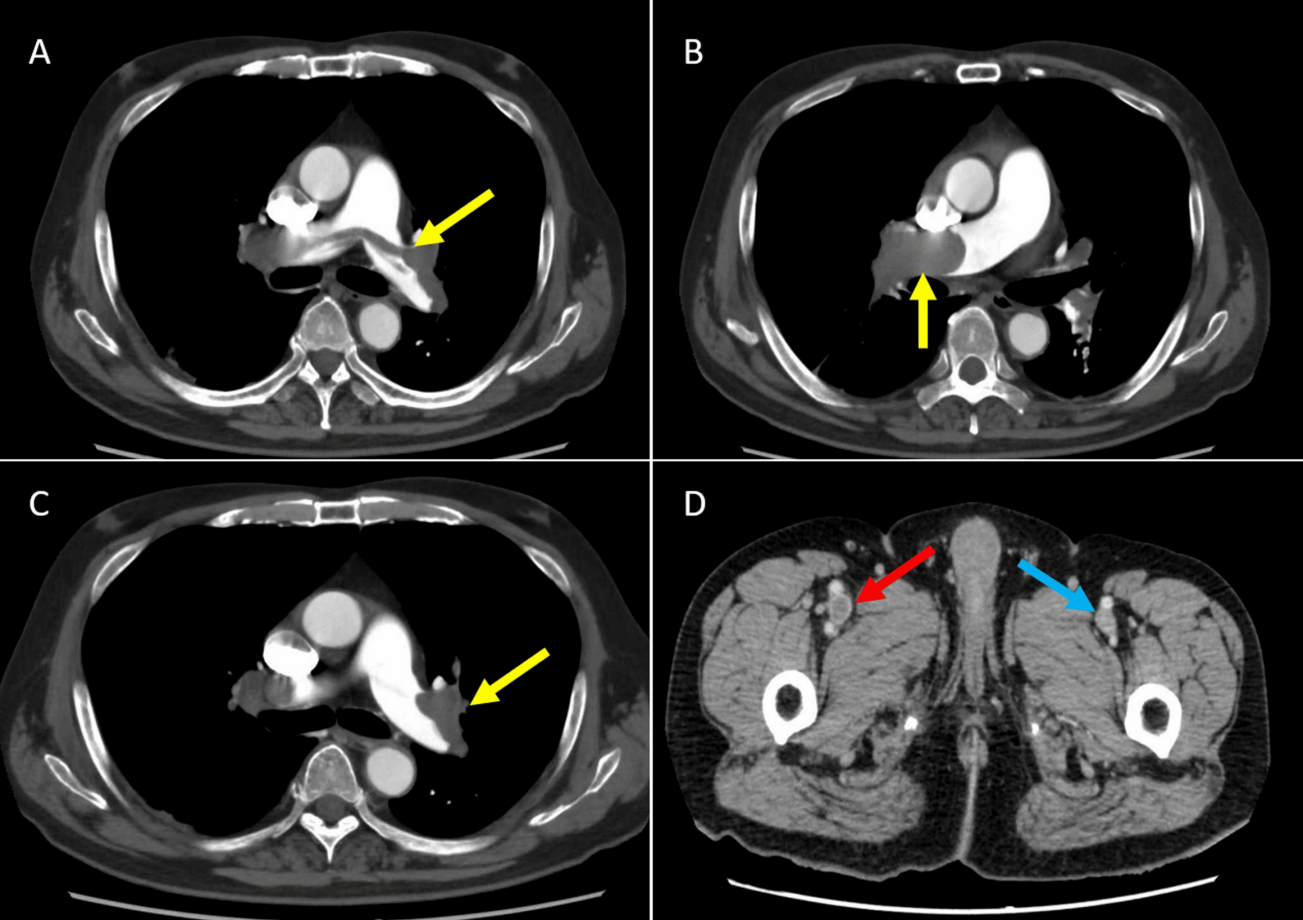
CT scan images at admission Contrast-enhanced CT images at presentation show evidence of acute pulmonary embolism and deep vein thrombosis. A, B, and C demonstrate filling defects within the pulmonary arteries (yellow arrows), consistent with bilateral thromboembolism. D shows a filling defect in the right femoral vein (red arrow), supporting a diagnosis of deep vein thrombosis, while no such defect was observed in the left femoral vein (blue arrow). These findings, alongside elevated D-dimer and right heart strain on echocardiography, confirmed systemic venous thromboembolism and prompted anticoagulation and thrombolytic therapy. CT, computed tomography

Contrast-enhanced computed tomography (CT) confirmed pulmonary embolism with extensive clot burden as well as right femoral vein thrombosis, although no pleural effusion was observed on the initial imaging (Figure 2).

**Figure 3:**
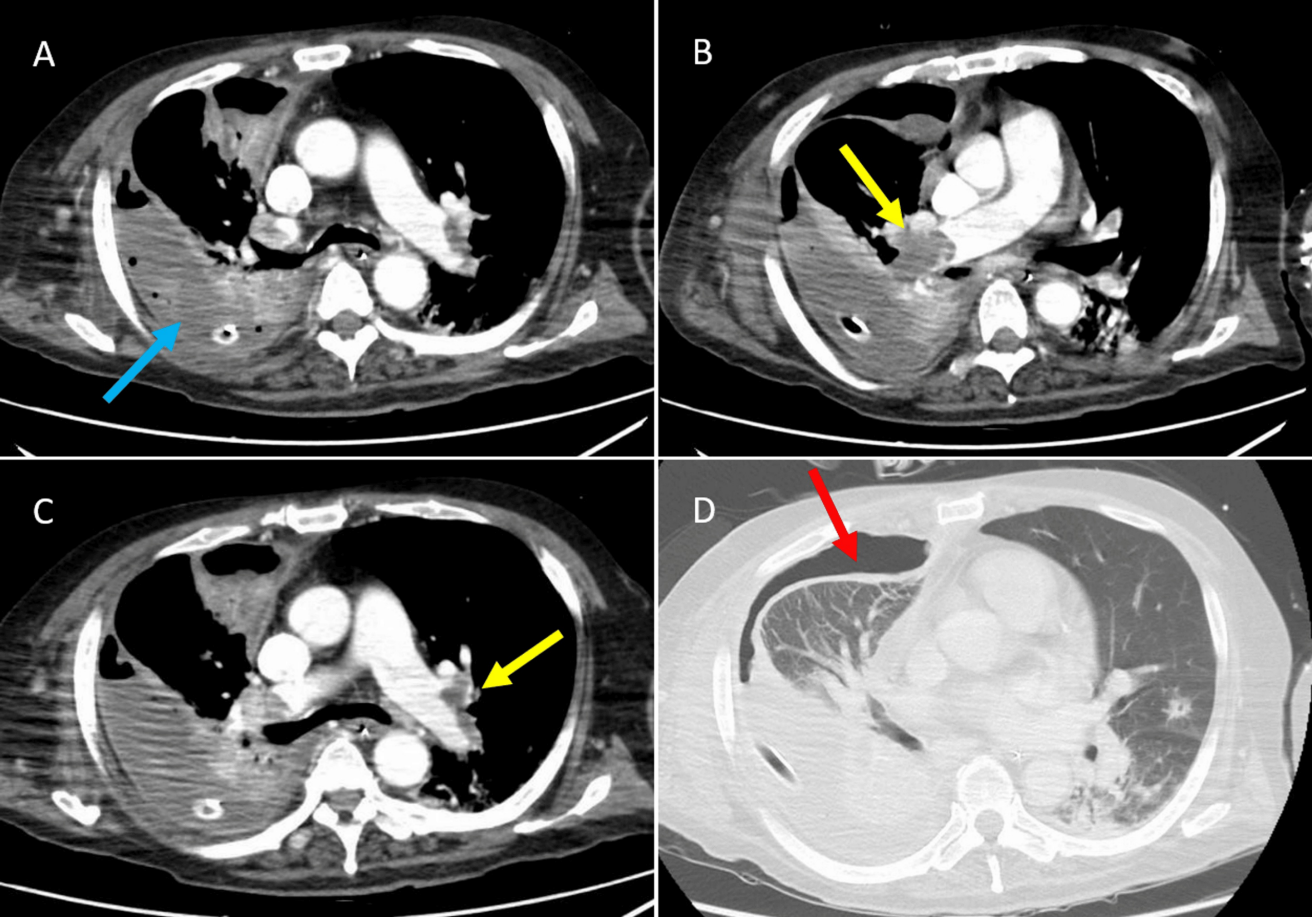
Follow-up CT images prior to saline irrigation CT images obtained on hospital day 21 illustrate complications secondary to anticoagulation and thrombolysis. A reveals a low-density area in the lower right lung (blue arrow), suggestive of empyema. B and C show persistent pulmonary artery thrombus (yellow arrows), and D displays a newly formed right-sided pneumothorax (red arrow). These findings corresponded with persistent fever, purulent drainage, and inadequate chest tube output, prompting the decision to initiate intrapleural saline irrigation due to the patient’s poor surgical candidacy. CT, computed tomography

He was admitted to the ICU and started on unfractionated heparin at a daily dose of 10,000 units, with the activated partial thromboplastin time (APTT) maintained at a target range of 1.5 to 2.5 times the control value (approximately 50 to 80 seconds). This regimen was continued for seven days, then resumed on day 14 after a brief interruption. He subsequently suffered a cardiac arrest, requiring VA-ECMO and administration of monteplase (mutant tPA). Nine hours later, he developed a massive right-sided hemothorax, necessitating the placement of a 24-Fr chest tube and massive transfusion, including 12 units of red blood cells, 22 units of fresh frozen plasma, and 10 units of platelets. Anticoagulation was resumed on day 7, and VA-ECMO was weaned by day 18. On day 21, purulent drainage was observed from the chest tube. Despite broad-spectrum antibiotics, fever ranged from 38.3 ℃ (101 ℉) to 39.5 ℃ (103 ℉), and elevated inflammatory markers persisted. Pseudomonas aeruginosa was isolated from both pleural drainage fluid and airway aspirates, further supporting the diagnosis of bacterial empyema. Follow-up CT showed persistent empyema, pneumothorax, and inadequate drainage. Pleural fluid biochemical analysis, including pH, LDH, and glucose, was not performed (Figure 3).

**Figure 4:**
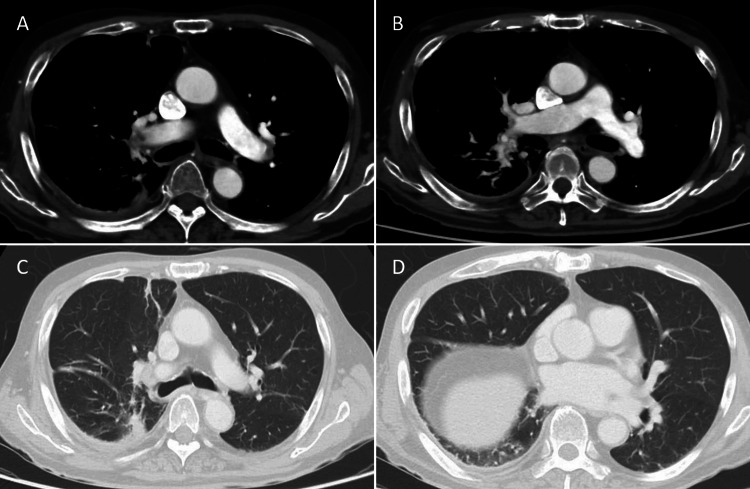
Follow-up CT images prior to patient transfer Follow-up CT scans performed prior to transfer of the patient show significant radiologic improvement following 32 days of saline irrigation. A and B demonstrate nearly complete resolution of the previously noted pulmonary embolism. C and D show marked improvement in pleural space condition, with no new fluid collections or evidence of pneumonia, correlating with cessation of purulent drainage and air leak. These results allowed for the successful discontinuation of oxygen and antibiotics, chest tube removal, and eventual patient transfer to a rehabilitation facility in stable condition. CT, computed tomography

The diagnosis of a bronchopleural fistula was based on two key findings: (1) the isolation of *Pseudomonas aeruginosa* with matching antimicrobial susceptibility profiles from both pleural drainage fluid and airway aspirates and (2) persistent pneumothorax and continuous air leak despite appropriate chest tube placement. These findings were further supported by CT on day 21, which demonstrated a communication between the pleural cavity and airway structures. Surgical intervention was deemed too risky due to his compromised condition and residual pulmonary embolism. A surgical tracheostomy was performed on day 21 using an 8.0 mm inner-diameter tube. On day 27, the chest tube was replaced with a 28-Fr double-lumen irrigation suction tube, and intrathoracic saline irrigation was initiated. The replacement procedure was performed under sterile conditions, and no signs of local infection or wound dehiscence were observed at the insertion site throughout the course. The same strain of *Pseudomonas aeruginosa* with matching antimicrobial susceptibility was consistently isolated from both pleural drainage fluid and airway aspirates during treatment, indicating persistent infection via a bronchopleural fistula rather than retrograde contamination. The drain was replaced through the original insertion site, and only a single chest drain was used at any time. Imaging confirmed appropriate drain positioning and the placement of additional drains was deemed unnecessary. This led to rapid resolution of fever and improved laboratory markers. Lavage was performed almost daily for 32 days, with saline volumes gradually increased from 20 mL to 250 mL per session, and up to 1,000 mL in divided doses on some days. Patient repositioning and intermittent suction were employed to optimize drainage. Occasional choking episodes occurred post-irrigation, but no saline was aspirated via the tracheostomy tube. Lavage was conducted using low pressure and under close monitoring; no signs of aspiration, respiratory compromise, or radiological evidence of lavage fluid entering the lung parenchyma were observed. On day 43, *Pseudomonas aeruginosa* was still isolated from the pleural drainage fluid. After that point, the drainage characteristics changed from purulent to clear yellow exudate, and the volume gradually decreased. Subsequent pleural fluid cultures were not obtained, and culture negativity could not be formally documented. By day 57, CT demonstrated significant improvement, with resolution of pneumothorax and reduction in abscess size. Air leakage completely ceased on day 58, and the chest tube was removed on day 62 following a successful clamping trial. Antibiotics were discontinued on day 89, based on the absence of fever for more than one week, sustained improvement in respiratory status, negative airway cultures, and normalization of inflammatory markers. A follow-up CT on day 155 showed near-complete resolution of the pulmonary embolism and no recurrence of pneumonia (Figure 4). The patient was transferred to a rehabilitation facility on day 177 and was later discharged home.

## Discussion

Empyema with a bronchopleural or alveolopleural fistula is an especially formidable clinical challenge, often associated with high morbidity and mortality due to the dual problems of persistent infection and abnormal communication between the pleural space and the airways. The presence of a bronchopleural or alveolopleural fistula complicates management by perpetuating air leaks, impairing pleural drainage, and increasing the risk of ongoing infection [2]. Conventional management for empyema typically involves prompt initiation of broad-spectrum antibiotics, nutritional support, and pleural drainage via tube thoracostomy. However, in the context of a fistula, these measures are frequently insufficient, necessitating escalation to more invasive interventions. Surgical options such as VATS, open thoracotomy with decortication, and OWT are often considered, but these procedures are highly invasive and may not be feasible in patients with significant comorbidities, poor respiratory reserve, or ongoing anticoagulation, as was the case in our patient.

Adjunctive intrapleural therapies have been developed to improve outcomes in empyema, with intrapleural fibrinolytics, such as streptokinase or tissue plasminogen activator (tPA) and DNase, showing benefit in select populations by facilitating drainage in loculated collections and reducing the need for surgery. However, these agents carry a risk of bleeding and are contraindicated in patients with active anticoagulation or recent thrombolytic therapy [3]. Furthermore, their efficacy in empyema complicated by fistula is not well established, and the literature remains limited on their use in this setting. In our case, the use of fibrinolytics was deemed unsuitable due to the patient’s recent administration of thrombolytic agents and ongoing anticoagulation, highlighting a common clinical dilemma.

Saline irrigation as an adjunct to tube thoracostomy has recently gained attention for its potential to enhance pleural drainage and infection control in empyema. The PIT, a pilot single-center randomized controlled trial conducted at Southmead Hospital, Bristol, UK, in 2015, demonstrated that pleural irrigation with normal saline led to a greater reduction in pleural fluid volume and a lower rate of surgical referral compared to standard care in patients with pleural infection [5]. Importantly, the technique was generally well tolerated, with few serious adverse events, and offers a practical solution in settings where surgery is not feasible [3]. However, most studies, including the PIT, excluded patients with bronchopleural or alveolopleural fistula due to concerns about the risk of saline entering the airways and causing aspiration pneumonia.

Despite these concerns, emerging evidence suggests that with careful monitoring and technique, saline irrigation may be beneficial even in the presence of a fistula. Reports using electrolyzed saline have described high rates of pleural disinfection, rapid improvement in inflammatory markers, and successful space closure without major complications [10,11]. Other studies have explored the use of intrapleural saline in combination with agents such as tyloxapol, further supporting its role as a viable adjunct in complex empyema cases [10,12].

While our approach utilized saline-only pleural irrigation via a double-lumen chest tube, recent protocols advocate for a more structured process incorporating intrapleural agents such as tyloxapol. An ideal protocol involves confirmation of complex pleural infection, exclusion of bronchopleural fistula, and image-guided insertion of a small-bore chest drain. Saline (250 mL of 0.9% NaCl) is instilled via gravity over one hour through a three-way tap, followed by a drainage phase and subsequent instillation of 200 mg tyloxapol diluted in 50 mL saline, which is clamped for 45 minutes. This cycle may be repeated every six to 12 hours based on clinical response [5,11,13]. Intrapleural saline irrigation is conditionally recommended when intrapleural TPA/DNase or surgery is unsuitable (BTS Guideline for Pleural Disease, 2023), supporting its role as an adjunctive or alternative approach [14]. Although our protocol did not include intrapleural tyloxapol, effective infection control and radiographic improvement were achieved, suggesting that saline-only irrigation may remain a practical alternative in selected cases, especially where resources or patient stability limit more complex interventions.

In our case, the decision to proceed with saline irrigation was driven by the patient’s inability to tolerate surgery due to a massive pulmonary embolism, recent cardiac arrest, and ongoing anticoagulation [9,15]. The irrigation protocol was individualized, starting with small volumes and gradually increasing as tolerated, with close monitoring for signs of aspiration or worsening air leak. Over a 32-day period, this approach led to rapid clinical improvement, resolution of fever and inflammatory markers, and radiological evidence of empyema cavity closure and fistula healing, without serious complications. Suction at 30 cmH₂O was intermittently applied to facilitate drainage, and the patient’s position was frequently changed to optimize fluid distribution and minimize the risk of aspiration [3,10]. The absence of pneumonia or other major complications in our patient is consistent with the safety profile reported in recent studies, provided that careful technique and vigilant monitoring are employed [11].

Recent advances in bronchoscopic techniques, such as endobronchial Watanabe spigot (EWS) placement, fibrin glue injection, and endoscopic stenting, offer additional options for fistula closure, especially in patients unfit for surgery [16,17]. These interventions can be combined with negative pressure wound therapy (NPWT) or saline irrigation to promote healing and infection control [18]. In a recent case, successful closure of a bronchopleural fistula was achieved using a comprehensive approach that included bronchial blocker placement, embolization, and intensive supportive care, underscoring the importance of a multidisciplinary strategy [19].

Comparative effectiveness studies and recent guidelines highlight that while surgery remains the gold standard for definitive management of empyema with fistula, non-surgical approaches, including prolonged drainage, intrapleural therapies, and minimally invasive procedures, can yield favorable outcomes in selected patients, particularly those at high operative risk [20]. The key to successful non-surgical management lies in careful patient selection, individualized protocol design, and close multidisciplinary oversight. Our case exemplifies how, in the absence of surgical options, a stepwise, protocol-driven approach to saline irrigation can achieve infection control and cavity closure, even in the context of a bronchopleural fistula [12,20].

It is important to acknowledge the limitations inherent to this approach and to our case. The evidence base for saline irrigation in empyema with fistula is still limited to small case series and retrospective studies, with a lack of large randomized controlled trials specifically addressing this population [11,12]. While the risk of pulmonary contamination from a pleural source is considered low in the reported series, it remains a theoretical concern and warrants further investigation. Additionally, the optimal volume, frequency, and duration of irrigation, as well as the role of adjunctive agents such as tyloxapol or electrolyzed saline, require further study [10,21]. Future research should focus on prospective, multicenter trials to define best practices and identify predictors of success for saline irrigation in this challenging population.

In summary, empyema with fistula is a complex and often life-threatening condition that traditionally necessitates aggressive surgical management. However, for patients who are poor surgical candidates, as illustrated in our case, intrapleural saline irrigation represents a promising alternative [9]. When performed with careful attention to technique, patient selection, and multidisciplinary support, this approach can achieve infection control and cavity closure without serious complications. Our experience, supported by emerging evidence, suggests that saline irrigation should be considered as part of the therapeutic armamentarium for complex empyema, particularly in high-risk patients where surgery is not feasible. Ongoing research and protocol refinement will be essential to optimize outcomes and expand the applicability of this minimally invasive strategy.

## Conclusions

This case demonstrates that intrapleural saline irrigation may serve as an effective and safe alternative to surgical intervention in patients with fistulous empyema who are poor surgical candidates. Individualized treatment strategies are essential, taking into consideration the availability of intrapleural medications, surgical risk, bleeding risk from fibrinolytic therapy, institutional resources and expertise, patient financial circumstances, and patient preferences. While this case highlights the promise of saline-only irrigation, further studies are warranted to evaluate the safety and efficacy of saline in combination with agents such as tyloxapol and to explore its potential as an alternative to DNase in conjunction with fibrinolytics. However, this report is inherently limited by its single-case design. Further prospective studies are needed to validate the reproducibility of this approach and to define standardized protocols regarding irrigation volume, duration, and agent selection. The findings from this report may serve as a foundation for future prospective studies aimed at optimizing non-surgical management strategies for complex empyema.

## References

[1] Light RW (2006). Parapneumonic effusions and empyema. Proc Am Thorac Soc.

[2] Sarkar P, Chandak T, Shah R, Talwar A (2010). Diagnosis and management bronchopleural fistula. Indian J Chest Dis Allied Sci.

[3] Rahman NM, Maskell NA, West A (2011). Intrapleural use of tissue plasminogen activator and DNase in pleural infection. N Engl J Med.

[4] Molnar TF (2007). Current surgical treatment of thoracic empyema in adults. Eur J Cardiothorac Surg.

[5] Hooper CE, Edey AJ, Wallis A (2015). Pleural irrigation trial (PIT): a randomised controlled trial of pleural irrigation with normal saline versus standard care in patients with pleural infection. Eur Respir J.

[6] Skrzypczak PJ, Kasprzyk M, Piwkowski C (2023). The review of the management and prevention methods of bronchopleural fistula in thoracic surgery. J Thorac Dis.

[7] Grapatsas K, Leivaditis V, Zarogoulidis P (2018). Successful treatment of postoperative massive pulmonary embolism with paradoxal arterial embolism through extracorporeal life support and thrombolysis. Respir Med Case Rep.

[8] Kearon C, Akl EA, Ornelas J (2016). Antithrombotic therapy for VTE disease: chest guideline and expert panel report. Chest.

[9] Lorusso R, Shekar K, MacLaren G (2021). ELSO interim guidelines for venoarterial extracorporeal membrane oxygenation in adult cardiac patients. ASAIO J.

[10] Nakamoto K, Takeshige M, Fujii T, Hashiyada H, Yoshida K, Kawamoto S (2016). Electrolyzed saline irrigation for elimination of bacterial colonization in the empyema space. Surg Infect (Larchmt).

[11] Guinde J, Laroumagne S, Chollet B, Trias-Sabrià P, Dutau H, Astoul P (2021). Saline lavage for the management of severe pleural empyema: a cohort study. Clin Respir J.

[12] He Z, Shen L, Xu W, He X (2020). Effective treatment of bronchopleural fistula with empyema by pedicled latissimus dorsi muscle flap transfer: two case report. Medicine (Baltimore).

[13] Mohamad Jailaini MF, Saini NA, Che Rahim MJ, Abdul Hamid MF (2024). A potential prospect: the novel treatment of intrapleural saline irrigation with intrapleural tyloxapol in treating thoracic empyema. Respirol Case Rep.

[14] Roberts ME, Rahman NM, Maskell NA (2023). British Thoracic Society Guideline for pleural disease. Thorax.

[15] Thiele H, Zeymer U, Neumann FJ (2012). Intraaortic balloon support for myocardial infarction with cardiogenic shock. N Engl J Med.

[16] Lois M, Noppen M (2005). Bronchopleural fistulas: an overview of the problem with special focus on endoscopic management. Chest.

[17] Date N, Komatsu T, Fujinaga T (2021). A novel technique for endobronchial Watanabe spigot placement for bronchopleural fistula: a traction method. Eur J Cardiothorac Surg.

[18] Harada A, Nakamura Y, Fukumori K, Nagata T, Iguro Y (2010). [Negative pressure wound therapy was useful in treating empyema with bronchopleural fistula]. Kyobu Geka.

[19] Monsch GM, Etienne H, Hillinger S, Caviezel C, Lauk O, Opitz I, Schneiter D (2024). Accelerated treatment concept in postpneumonectomy empyema with bronchopleural fistula. Sci Rep.

[20] Mao R, Ying PQ, Xie D (2016). Conservative management of empyema-complicated post-lobectomy bronchopleural fistulas: experience of consecutive 13 cases in 9 years. J Thorac Dis.

[21] Puskas JD, Mathisen DJ, Grillo HC, Wain JC, Wright CD, Moncure AC (1995). Treatment strategies for bronchopleural fistula. J Thorac Cardiovasc Surg.

